# Gene expression is stable in a complete *CIB1* knockout keratinocyte model

**DOI:** 10.1038/s41598-020-71889-9

**Published:** 2020-09-11

**Authors:** Elias Imahorn, Magomet Aushev, Stefan Herms, Per Hoffmann, Sven Cichon, Julia Reichelt, Peter H. Itin, Bettina Burger

**Affiliations:** 1 Department of Biomedicine, University of Basel and University Hospital Basel, Basel, Switzerland; 2 Wellcome Centre for Mitochondrial Research, Institute of Genetic Medicine, Newcastle upon Tyne, UK; 3 Institute of Human Genetics, Division of Genomics, Life & Brain Research Centre, University Hospital of Bonn, Bonn, Germany; 4 Institute of Neuroscience and Medicine (INM-1), Genomic Imaging, Research Center Juelich, Juelich, Germany; 5 Department of Dermatology, Venereology and Allergology, Medical University Innsbruck, Innsbruck, Austria; 6 Department of Dermatology, University Hospital Basel, Basel, Switzerland

**Keywords:** Genetics, Molecular biology, Diseases, Pathogenesis

## Abstract

Epidermodysplasia verruciformis (EV) is a genodermatosis characterized by the inability of keratinocytes to control cutaneous β-HPV infection and a high risk for non-melanoma skin cancer (NMSC). Bi-allelic loss of function variants in *TMC6, TMC8,* and *CIB1* predispose to EV. The correlation between these proteins and β-HPV infection is unclear. Its elucidation will advance the understanding of HPV control in human keratinocytes and development of NMSC. We generated a cell culture model by CRISPR/Cas9-mediated deletion of *CIB1* to study the function of *CIB1* in keratinocytes. Nine *CIB1* knockout and nine mock control clones were generated originating from a human keratinocyte line. We observed small changes in gene expression as a result of *CIB1* knockout, which is consistent with the clearly defined phenotype of EV patients. This suggests that the function of human CIB1 in keratinocytes is limited and involves the restriction of β-HPV. The presented model is useful to investigate CIB1 interaction with β-HPV in future studies.

## Introduction

Epidermodysplasia verruciformis (EV) (OMIM 226400) is a primary immunodeficiency to cutaneous human papillomaviruses of the genus β-HPV^1–3^. Furthermore, patients with clinical symptoms of EV associated with inborn or acquired immunodeficiency by T-cell defects have been reported. This clinical picture is referred to as “atypical EV”^1^. Patients with congenital EV exhibit plane warts with onset in early childhood and have an increased risk of developing non-melanoma skin cancer (NMSC)^3–5^. More than 500 patients have been described to date^6^. Bi-allelic pathogenic variants in *TMC6* or *TMC8* (also known as *EVER1* and *EVER2*, respectively) have been identified in 22 families (Table 1). Recently, we identified homozygous loss-of-function variations in *CIB1* in six EV-families^7^. Majority of the bi-allelic loss-of-function variants in these three genes are either nonsense, frameshift, or splice site variants. A homozygous three amino acid in-frame duplication in *TMC8* was recently described in one family^8^. Loss of protein expression has been shown in patients with a frameshift in *TMC8*^9^ and in patients with *CIB1* variants^7^.

**Table 1:** In 28 families, 26 different EV-causing variants in *TMC6, TMC8,* and *CIB1* have been described.

Disease-causing variant	Gene	Type	Patients	Families	References
c.220C>T	*TMC6*	Nonsense	1	1	Aochi et al.^50^
c.280C>T	*TMC6*	Nonsense	5	2	Ramoz et al.^17^
c.[744C>A];[892-2A>T]	*TMC6*	Nonsense/splice site	1	1	Tate et al.^51^
c.892-2A>T	*TMC6*	Splice site	1	1	Sunohara et al.^52^
c.916_917insCATGT	*TMC6*	Frameshift	2	1	Zuo et al.^53^
c.968delT	*TMC6*	Frameshift	1	1	Gober et al.^54^
c.1110C>G	*TMC6*	Nonsense	2	1	Youssefian et al.^8^
c.1726G>T	*TMC6*	Nonsense	3	1	Ramoz et al.^17^
c.188G>A	*TMC8*	Nonsense	3	1	Rady et al.^55^
c.326_338del	*TMC8*	Frameshift	1	1	Landini et al.^9^
p.Thr150fs*3	*TMC8*	Frameshift	3	1	Lazarczyk et al., Heuser et al.^11,56^
c.561_583del	*TMC8*	Frameshift	1	1	Berthelot et al.^57^
c.568C>T	*TMC8*	Nonsense	1	1	Sun et al.^58^
c.571delG	*TMC8*	Frameshift	1	1	Landini et al.^9^
c.755delT	*TMC8*	Frameshift	1	1	Ramoz et al.^17^
c.1084G>T	*TMC8*	Nonsense	3	1	Ramoz et al.^17^
c.1127 + 1G>C	*TMC8*	Splice site	3	1	Imahorn et al.^6^
c.1233C>A	*TMC8*	Nonsense	1	1	Youssefian et al.^8^
c.1477_1485dup	*TMC8*	In frame duplication	1	1	Youssefian et al.^8^
c.1534-3_1534-2delCA	*TMC8*	Splice site	1	1	Miyauchi et al.^59^
c.1824-1G>A	*TMC8*	Splice site	1	1	Mizuno et al.^60^
c.52-2A>G	*CIB1*	Splice site	5	1	de Jong et al.^7^
c.214C>T	*CIB1*	Nonsense	4	1	de Jong et al.^7^
c.248_249delAA	*CIB1*	Frameshift	3	2	de Jong et al.^7^
c.465_465 + 1insG	*CIB1*	Frameshift	11	1	de Jong et al.^7^
c.549_550insTT	*CIB1*	Frameshift	1	1	de Jong et al.^7^

CIB1, TMC6, and TMC8 form a complex and CIB1 has been shown to be degraded in case of one missing interaction partner^7^. The CIB1-TMC6-TMC8 complex acts as restriction factor for β-HPV via an unknown mechanism. Effects based on known functions of these three proteins were disproven in patients’ keratinocytes^7^. CIB1 has been shown to influence cell migration and adhesion in animal models^10^. These processes normally work in *CIB1* deficient patients’ keratinocytes^7^. It has been hypothesized that TMC6 and TMC8 influence intracellular zinc levels, which control activity of transcription factors such as AP-1, which is important for the HPV life cycle^11,12^. However, changes in intracellular zinc homeostasis were not observed in TMC6, TMC8, or CIB1 deficient keratinocytes derived from EV patients^7^. Finally, TMC8 was hypothesized to switch the cellular response to TNF-α from pro-apoptotic to pro-survival combined with NF-κB activation^13,14^. Such changes could not be confirmed in keratinocytes from EV patients or in in vitro studies^7^. Additionally, no influences on the transcriptome could be identified in the patients' cells by RNA-seq or qPCR. Due to the heterogeneity of patients' samples, small differences may be lost in the background. The presented keratinocyte model aimed to overcome this diversity and to identify effects of *CIB1* deficiency on the expression of downstream genes.

## Materials and methods

### Cell culture and validation of keratinocyte line

The human keratinocyte line NKc21^15^ was maintained in CnT-PR medium (CELLnTEC Advanced Cell Systems, Bern, Switzerland). The mouse fibroblast line 3T3^16^ was maintained in DMEM (Lonza, Basel, Switzerland) supplemented with 10% FCS (Thermo Fisher Scientific, Waltham, MA, USA). All cell lines were cultured at 37 °C in a 5% CO_2_ atmosphere.

*CIB1, TMC6,* and *TMC8* genes of NKc21 were analysed by Sanger sequencing after PCR amplification from isolated gDNA. Sequences of primers for *TMC6* and *TMC8*^17^ were kindly provided by Gérard Orth and Michel Favre (Department of Virology, Institute Pasteur, Paris, France). Primer sequences for *CIB1* are listed in Table S1. Expression of *TMC6, TMC8,* and *CIB1* was verified by RT-PCR on RNA isolated from NKc21 using primers CIB1_9, CIB1_10, TMC6_1, TMC6_2, TMC8_1, and TMC8_2 (Table S1). Additionally, gDNA was analyzed by an Illumina HumanOmniExpressExome-8 BeadChip v1.3 SNP array. SNP array data was visualized using GenomeStudio and copy number analysis performed using CNV-Partition (both Illumina, San Diego, CA, USA).

### Transfection of keratinocytes

Transfection of keratinocytes was performed using Xfect transfection reagent (Takara Bio, Kusatsu, Japan) as previously described^18^. In short, 1 day after seeding 70,000 cells per well in 6-well plates, the medium was replaced by fresh medium and a mixture of 330 ng of each CRISPR/Cas9 plasmid (total of 660 ng DNA), 0.2 µl Xfect polymer, and 100 µl Xfect buffer was added to the keratinocytes after incubation at room temperature for 10 min. After four hours at 37 °C, the medium was replaced by 3 ml fresh CnT-PR.

### Generation of active CRISPR plasmids

Sequences for sgRNAs were chosen based on the minimal off-target activity predicted by an online tool (https://crispr.mit.edu)^19,20^. Plasmids expressing Cas9 and sgRNA were generated by inserting custom sgRNA sequences into pSpCas9(BB)-2A-GFP (Addgene, Cambridge, MA, USA, #48138) as described^20^. Plasmids and sgRNA used in this study are listed in Table S2. After treatment with ATP-dependent DNase (Lucigen, Middleton, WI, USA), DH5α chemo-competent *E. coli* were transformed and selected in the presence of ampicillin (Thermo Fisher Scientific). Correct insertion of the sgRNA was verified by sequencing of isolated plasmids (Nucleospin plasmid kit, Macherey–Nagel, Düren, Germany) using U6 primer.

### Validation of CRISPR/Cas9 plasmids

**Figure 1:**
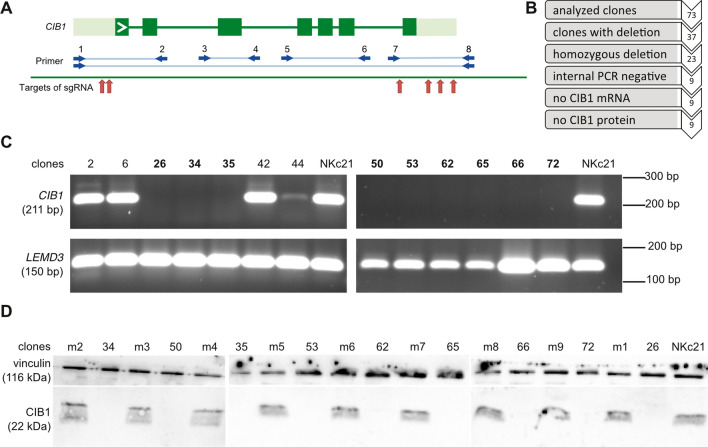
Nine keratinocyte clones showed a knockout of *CIB1* without any detectable transcript or protein product. (**A**) Primers CIB1_1–CIB1_8 were used to amplify regions of *CIB1* or the whole gene. Locations of primers and the resulting amplicons (blue lines and arrows) respective to *CIB1* and target sites of sgRNAs (red arrows) are depicted. (**B**) Flowchart shows the screening process to identify successfully edited clones. Nine clones out of 73 had a complete homozygous *CIB1* deletion. (**C**) Expression of *CIB1* mRNA in various clones and untreated NKc21 was checked by RT-PCR. *LEMD3* was used as a positive control. Nine clones (bold numbers) showed no amplification of *CIB1* transcript. The uncropped gel pictures can be found in Figure S3. (**D**) Presence of CIB1 protein was analysed by Western blot in the nine *CIB1*^−/−^ clones, nine mock transfected clones (m1–m9), and untreated NKc21. Vinculin was used as loading control. All nine *CIB1*^−/−^ clones showed complete absence of CIB1 protein while all mock transfected control clones and untreated NKc21 keratinocytes retained CIB1 expression. The uncropped blot pictures can be found in Figure S4.

Deletion activity of five combinations of sgRNAs with target sites on both sides of *CIB1* was tested. For this, NKc21 were transfected with plasmids encoding Cas9 and the tested sgRNAs. Six days after transfection, gDNA was isolated and the target region was amplified using primer CIB1_1 and CIB1_8 (Table S1) located outside of the intended deletion (Fig. 1A). This PCR yields a 4,130 bp wild-type amplicon and in case of successful deletion smaller additional amplicons (in the size range of 500–700 bp).

### Generation of cell line models

Combinations of plasmids were transfected into NKc21 keratinocytes (Table 2). After 5 days, single GFP-positive cells were sorted into 96-well plates pre-seeded with 3T3 murine fibroblasts previously growth arrested with 4 mg l^−1^ mitomycin C (StressMarq Biosciences, Cadboro Bay, Canada) as described^18^. Single cell sorting was performed on a FACS Aria III cell sorter (BD Biosciences, Franklin Lakes, NJ, USA) using 100 µm nozzle and 15 psi pressure. Clones were expanded from single cells and serially transferred to 24-well plates, T25, and T75 cell culture flasks. During this process, a few cells of each clone were transferred to 20 µl dilution buffer by scratching with a pipet tip. To lyse cells, 0.5 µl DNA release additive was added and the mixture incubated at room temperature for 5 min and at 95 °C for 2 min. These samples were used to screen clones for successful homozygous knockout of *CIB1* using Phire Tissue Direct PCR kit according to manufacturer’s instructions (Thermo Fisher Scientific) with primer combinations CIB1_1/CIB1_8, CIB1_3/CIB1_4, and CIB1_5/CIB1_6 (Table S1). Clones with successful *CIB1* knockout were harvested and split into four samples. Two samples were stored in liquid nitrogen for subsequent cell culture experiments. The other two samples were pelleted by centrifugation at 400 g at room temperature for 10 min. One pellet was frozen at − 20 °C for DNA isolation, the other was resuspended in 100 µl RA1 buffer and 2 µl TCEP from the NucleoSpin RNA XS kit (Macherey–Nagel) for RNA isolation.

**Table 2:** Genotype of *CIB1*^−/−^ clones used in the study.

Clone	sgRNA combination	Status of introduced alteration	Deletion in *CIB1* (NM_006384.3)
clone_26	CIB1-sgR-2/CIB1-sgR-3	Homozygous	c.-189_555-8del
clone_34	CIB1-sgR-1/CIB1-sgR-3	Compound heterozygous	c.[-188_549del]; [-201_*47del]
clone_35	CIB1-sgR-1/CIB1-sgR-3	Homozygous	c.-189_555-1delinsC
clone_50	CIB1-sgR-2/CIB1-sgR-5	Compound heterozygous, additional heterozygous duplication	c.[-195_*111del ; *198_*203dup]; [-188_*78del]
clone_53	CIB1-sgR-2/CIB1-sgR-5	Homozygous	c.-310_*119del
clone_62	CIB1-sgR-2/CIB1-sgR-5	Homozygous	c.-188_*78del
clone_65	CIB1-sgR-2/CIB1-sgR-5	Homozygous	c.-189_*78del
clone_66	CIB1-sgR-2/CIB1-sgR-5	Homozygous	c.-188_*78del
clone_72	CIB1-sgR-2/CIB1-sgR-5	Homozygous	c.-188_*78del

For generation of control clones, NKc21 were transfected with mock plasmids encoding Cas9 and sgRNA without target sequences in the human genome (mock-sgR-1, mock-sgR-2, and mock-sgR-3; Table S2). Single cell sorted clones were grown and harvested in the same way as the *CIB1* knockout clones.

### Evaluation of clones

Isolation of gDNA from keratinocytes was performed using the NucleoSpin tissue kit (Macherey–Nagel) according to the manufacturer’s instructions for cultured cells. Isolation of RNA from keratinocytes was performed according to the manufacturer’s instructions for RNA purification from cultured cells using the NucleoSpin RNA XS kit, but without addition of poly-A carrier RNA to not impair RNA-seq. Concentrations of isolated nucleic acids were determined using a DS-11 spectrophotometer (DeNovix Wilmington, DE, USA) and a Qubit fluorometer (Thermo Fisher Scientific). *CIB1* deletion was validated on isolated gDNA with PCR using Taq polymerase (Qiagen, Venlo, Netherlands) and the same primer combinations as for the Direct PCR during screening. Additionally, the amplicons covering the targeted region (primers CIB1_1/CIB1_8) were analysed by Sanger sequencing (Microsynth, Balgach, Switzerland) to detect the exact junction sites of deletion. To prove the lack of *CIB1* RNA expression, RNA isolated from the clones and the original NKc21 keratinocytes was reverse-transcribed using Verso cDNA synthesis kit (Thermo Fisher Scientific) according to manufacturer’s instructions using a 3:1 mixture of poly-A primers and random hexamers. Subsequently, RT-PCR was performed for *CIB1* (primer CIB1_9 and CIB1_10).

### Western blot

Western blot was performed to confirm CIB1 knockout on protein level. Keratinocytes were grown in T75 cell culture flasks and lysed in radio-immunoprecipitation (RIPA) buffer supplemented with protease and phosphatase inhibitor cocktail (Thermo Fisher Scientific) on ice; protein concentration was quantified using Pierce BCA protein assay (Thermo Fisher Scientific). 7.5 µg protein lysate per lane in Laemmli buffer were separated via SDS-PAGE on a 20% polyacrylamide gel and protein was transferred for 60 min at 350 mA onto nitrocellulose membrane. Polyclonal chicken anti-CIB1 antibody raised against amino acids 24–43^21,22^ was used at a dilution of 1:2,500. Monoclonal rabbit anti-vinculin antibody was used as loading control (Abcam, Cambridge, UK, ab129002, 1:10,000). Polyclonal goat HRP-conjugated antibodies to chicken IgY (Abcam, ab6877, 1:10,000) and rabbit IgG (Lifespan Biosciences, Seattle, WA, USA, LS-C60884, 1:3,000) were used as secondary antibodies. Signal was detected with SuperSignal West Pico Chemiluminescent Kit (Thermo Fisher Scientific) using a ChemiDoc SRX imager system (BioRad Laboratories, Hercules, CA, USA).

### RNA-seq

Gene expression of the nine *CIB1* knockout clones was compared to the nine mock transfected clones by RNA-seq. RNA quality of six samples was analyzed on a 2100 BioAnalyzer (Agilent Technologies, Santa Clara, CA, USA) with the RNA 6000 Nano kit (Agilent Technologies) and all six samples had an RNA integrity number (*RIN*) between 9.6 and 10^23^. Nine replicates per group with a sequencing depth of 10 million reads per sample are considered powerful for this type of experiment^24,25^. Libraries were prepared using QuantSeq 3′ mRNA-Seq Library Prep Kit FWD for Illumina (Lexogen, Vienna, Austria) and sequenced on a HiSeq2500v4 instrument (Illumina) in standard 3′ single read rapid mode for 50 cycles. Library preparation and sequencing was performed by the NGS Core Facility of the Life & Brain Center of the University of Bonn. Quality control, mapping, and counting was performed by the integrated data analysis pipeline on Bluebee platform (Bluebee Holding, Rijswijk, Netherlands) using FASTQC (Babraham Institute, Cambridge, UK), STAR^26^, and HTSeq-count^27^. Filtering, normalization, and differential gene expression analysis was performed using edgeR^28^: Genes with fewer than 1 count per million in over 9 samples were not included in further analysis (removing 78.21% of the genes but only 0.27% of the counts). Normalization was done by calculating a scaling factor for each sample resulting in the lowest possible log-fold changes between samples using trimmed mean of M-values^29^. The scaling factors ranged from 0.953 to 1.072. Common, trended, and tag-wise dispersion was calculated using the quantile-adjusted conditional maximum likelihood method (qCML)^30,31^. Genes differentially expressed in the knockout clones compared to the mock transfected control clones were determined using an exact test^30^. Genes with a false discovery rate (*FDR*) < 0.05 were considered differentially expressed. To validate the results, all steps were repeated with CLC Genomics Workbench (Qiagen) according to vendor’s instructions. Sequence reads of the RNA-seq experiment can be accessed in the European Nucleotide Archive (ENA) under the accession number "PRJEB34355".

### Validation of RNA-seq results by qRT-PCR

Expression of genes, that were differentially expressed with an *FDR* < 0.05 as calculated with either edgeR or CLC Genomics Workbench, and three housekeeping genes (*GUSB, HPRT1,* and *TBP*) was determined by qRT-PCR to validate the RNA-seq results. First, RNA samples were reverse transcribed using Verso cDNA synthesis kit (Thermo Fisher Scientific) according to manufacturer’s instructions. Each 20 µl reaction contained 10 µl Power SYBR Green PCR Master Mix (Thermo Fisher Scientific), 100 nM of each primer, and template cDNA corresponding to 12.5 ng RNA. For qPCR, cDNA was denatured at 95 °C for 10 min and amplified during 45 cycles using standard protocol in a 7500 Fast Real-Time PCR system (Thermo Fisher Scientific). Primer specificity was determined by melt curves. All reactions were performed in triplicates. Gene expression was normalized and quantified using qbase + (Biogazelle, Gent, Belgium). Confidence intervals were calculated using a Mann–Whitney U test.

## Results

### Validation of cell line

The human keratinocyte line NKc21^15^ was genetically analyzed to confirm its suitability for the presented project. Sanger sequencing of *CIB1*, *TMC6*, and *TMC8* did not reveal loss of function variants and only frequent (*MAF* > 0.05) heterozygous SNPs (rs12449858, rs145016347, and rs7208422) were found. Normal expression level of the three genes was verified by RT-PCR. SNP array of gDNA revealed six numerical aberrations (Fig. S1): trisomy of chromosomes 5, 8q, 18, and 20; monosomy of chromosome 8p; and trisomy of chromosome 9 in a subpopulation of cells. Neither chromosomes 15 nor 17 containing the EV-causing genes showed numerical aberrations in SNP array. Therefore, the cells were suitable for genome editing and subsequent downstream analyses investigating the consequences of CIB1 depletion on gene expression.

### Deletion activity of sgRNA

CRISPR/Cas9 was used with pairs of sgRNAs targeting both the 5′- and 3′- end of *CIB1*, to cause a deletion of the sequence between the target sites containing the complete gene. Four pairs of custom sgRNAs targeting both sides of the *CIB1* gene were tested for their function in NKc21. Amplification of the entire *CIB1* gene revealed a PCR product shortened by the intended length of ~ 3,500–3,700 bp depending on the pair used (Fig. S2). This experiment revealed three pairs of sgRNAs (1&3, 2&3, and 2&5) with sufficiently high activity for further use.

### Generation of cell line model

In order to generate multiple *CIB1* knockout cell lines, 73 clones were cultured from single NKc21 cells after genome editing. Successful *CIB1* knockout was confirmed by PCR (Fig. 1A). In a first reaction (primers 1 & 8), the whole gene was amplified. A shortened amplicon, indicating the successful deletion of *CIB1*, was produced in 36 clones, 13 of these were heterozygously and 23 clones homozygously deleted (Fig. 1B). The second PCR (primers 5 & 6) targeted a region (exons 4–6) within *CIB1*, confirming a genome-wide deletion of *CIB1* by absence of transcript in nine clones (Fig. 1C). Therefore, these nine clones were classified as complete *CIB1* knockout clones and characterized in more detail. A PCR product spanning both sgRNA recognition sites was sequenced to locate the exact break points of the deletion. This revealed deletion of the complete *CIB1* gene with break points near the sgRNA recognition sites (Table 2). The *CIB1* protein was not detectable in any of these clones (Fig. 1D).

To obtain control cell lines with a similar genetic background, nine mock transfected and single cell sorted clones were generated in the same way as the knockout clones. CIB1 protein expression was confirmed in these clones by Western blot (Fig. 1D).

### RNA-seq

**Figure 2:**
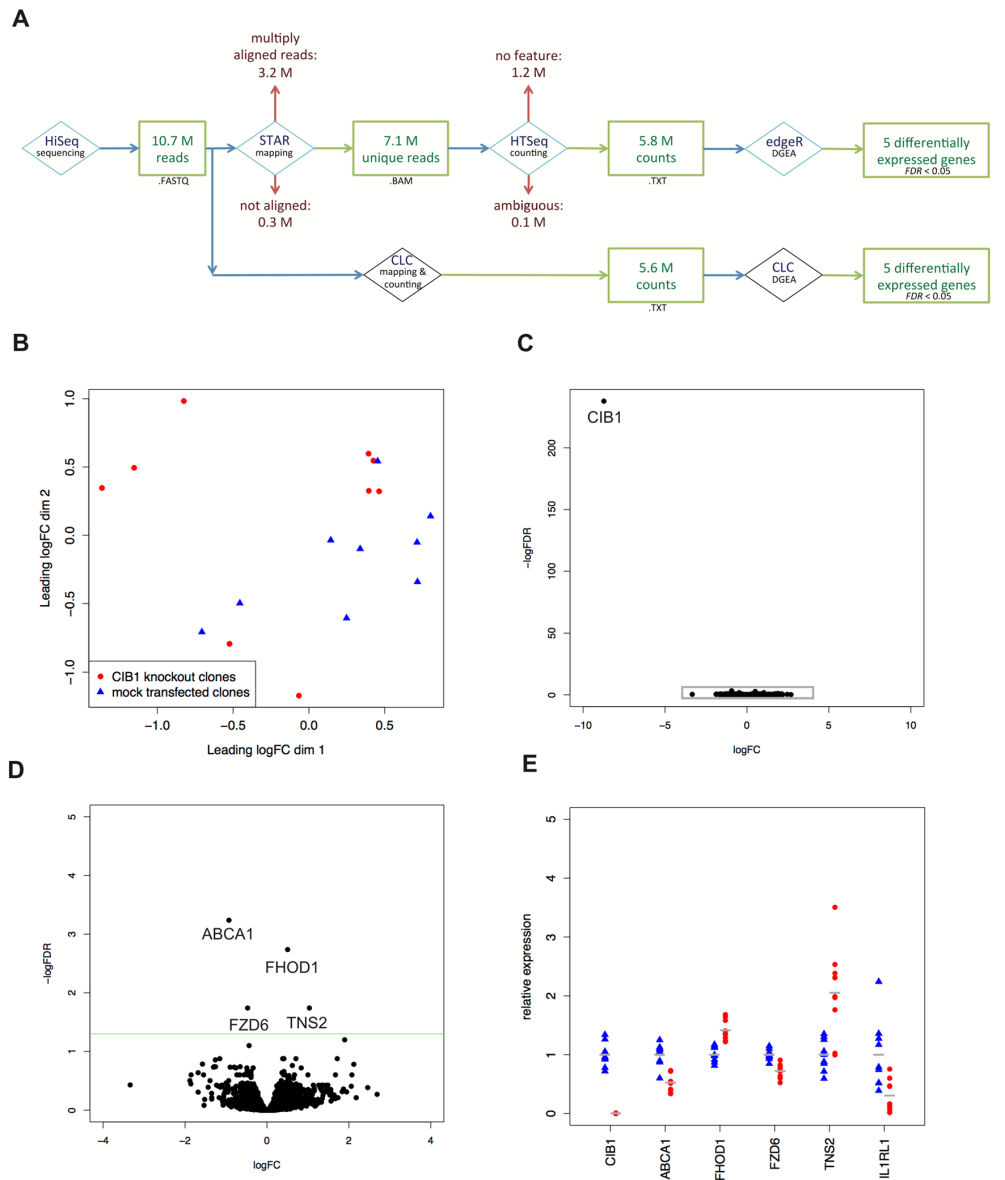
Differences in gene expression between the nine CIB1 knockouts and nine mock transfected clones were revealed using RNA-seq. (**A**) Workflow for analysis of RNA-seq data by STAR, HTSeq, and edgeR as well as CLC Genomics Workbench. The average number of remaining million reads after each calculation step are indicated. (**B**) MDS plot of all 18 samples by edgeR revealed small differences in gene expression caused by *CIB1* knockout. (**C**) The Volcano plot shows difference in gene expression between *CIB1* knockout clones and mock transfected clones. The gray box marks the area that is enlarged in (**D**). (**D**) Enlarged Volcano plot without *CIB1* showing four differentially expressed genes above the threshold of *FDR* = 0.05 (green line). (**E**) Relative expression of genes differentially expressed as calculated by edgeR. Expression has been normalized to the average count per million of the nine control clones. Mock clones are depicted in blue and knock-out clones in red. Each data point is an independent clone.

Transcriptome analyses by RNA-seq enabled the detection of differences in gene expression between the *CIB1* ko clones and control clones. On average, 10.7 million reads were obtained per sample. After discarding reads that aligned to multiple regions, 7.1 million with a unique alignment in the human genome were retained (Fig. 2A). Per sample, 5.8 million reads were assigned to a transcript. MDS plot showed only small differences between knockout and control clones and the samples did not cluster by group (Fig. 2B). Differential gene expression analysis calculated by edgeR and CLC Genomic Workbench resulted in a total of six transcripts with an *FDR* < 0.05 (*ABCA1, CIB1, FHOD1, FZD6, IL1RL1,* and *TNS2*) (Table 3), four of them identical for both algorithms (*ABCA1, CIB1, FHOD1,* and *TNS2*) (Fig. 2C,D). While *CIB1* expression was reduced more than 500-fold, the fold-changes of the other transcripts ranged between 0.178 and 2.07 (Fig. 2E).

**Table 3:** Average count per million (CPM), false discovery rate (FDR), and fold changes obtained by RNA-seq as well as fold changes and confidence interval (calculated by Mann–Whitney U test) obtained by confirmatory qRT-PCR.

Gene	CPM (RNA-seq)	FDR (edgeR)	Fold change (edgeR)	FDR (CLC)	Fold change (CLC)	Fold change qRT-PCR (95% CI)
*CIB1*	48.17	7.48E−244	0.00234	4.70E−238	0.00236	Knockout^a^
*IL1RL1*	6.75	0.229	0.313	0.0166	0.178	0.212 (0.077–0.585)
*ABCA1*	69.70	0.000468	0.524	0.0000649	0.518	0.551 (0.385–0.787)
*FZD6*	87.18	0.0154	0.719	0.0809	0.719	0.757 (0.333–1.720)
*FHOD1*	246.77	0.00136	1.42	0.0166	1.42	1.320 (1.110–1.568)
*TNS2*	15.61	0.0154	2.05	0.0159	2.07	2.150 (1.367–3.379)

^a^No amplicon was produced by RT-PCR in the knockout clones (see Fig. 1C).

### Validation of RNA-seq results by qRT-PCR

RNA-seq results were verified by qRT-PCR using the same samples. After normalization with three housekeeping genes (*GUSB, HPRT1,* and *TBP*) relative expression of *ABCA1*, *FHOD1*, *FZD6*, *IL1RL1*, and *TNS2* was quantified. qRT-PCR confirmed the fold-changes observed with RNA-seq. Differences in gene expression between *CIB1* knockout clones and mock transfected clones were significant for *ABCA1, FHOD1, TNS2,* and *IL1RL1* as calculated using the Mann–Whitney *U* test (Table 3).

## Discussion

**Figure 3:**
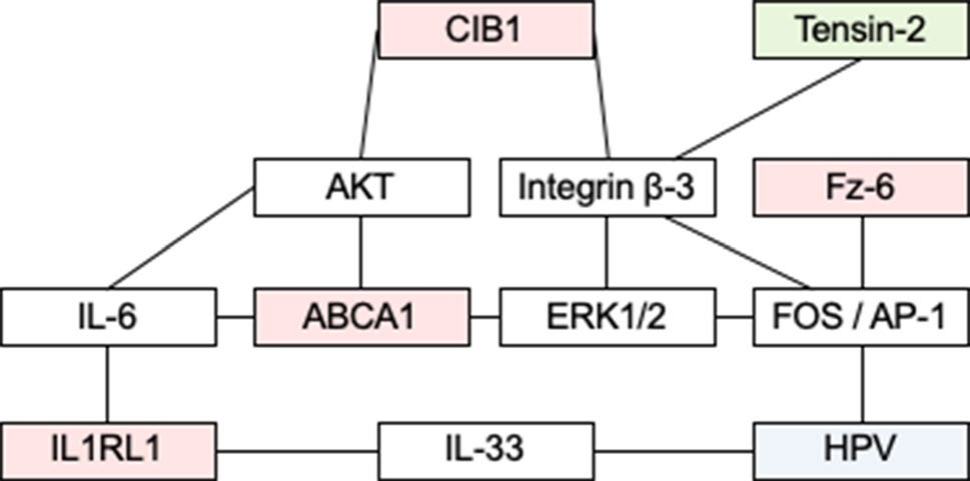
Known regulatory relationships between differentially expressed genes and their correlation to HPV. Network shows the links discussed in the literature between the differentially expressed genes and *CIB1*. Transcripts which were upregulated in the *CIB1*^−/−^ clones in the presented study are depicted in green, downregulated transcripts in red.

The presented study on a keratinocyte line with a complete *CIB1* knockout showed a slight effect of *CIB1* deficiency on expression of other genes. RNA-seq analysis of a high number of samples with an isogenic background revealed small changes in few genes. These identified low-level changes in single genes confirm the limited influence of CIB1 in keratinocytes. This is in accordance with expression data from keratinocytes of *CIB1* deficient EV-patients, which showed small differences in the expression of only 40 genes^7^. No enrichment of a specific pathway was observed. It is to be expected that CIB1 and both TMC influence similar or even the same pathways as they form a complex and the stability of CIB1 is dependent on TMC8 at the least^7^. On the other hand, CIB1 deficiency has no effect on expression of TMC6 and TMC8^7^, which is consistent in the presented data. TMC8 has been hypothesized to control β-HPV by promoting TNF-α induced apoptosis. Variants in TMC8 lead to NF-κB activation and pro-survival as response to TNF-α^13^. However, no indications for changed regulation of downstream targets of TNF-α or NF-κB have been identified in the presented or previous studies^7^, giving rise to the suspicion that CIB1/TMC6/TMC8 complex restricts β-HPV by a different mechanism. Fold-changes of the significantly differential expressed genes *ABCA1, FZD6, IL1RL1, TNS2,* and *FHOD1* were small and these genes could only indirectly be connected to innate immunity to β-HPV (Fig. 3)^22,32–43^. Future studies might show whether these proteins play a relevant role in the pathomechanism of EV.

EV patients with CIB1 deficiency have a clearly defined phenotype including the high susceptibility to β-HPV, development of plane warts, and the increased risk of developing NMSC. This suggests that the function of CIB1 in keratinocytes is the control of specific HPV, which would subsequently promote the development of NMSC^7^. No additional features shared by a significant subset of patients have been observed in many well documented patients^6,44–47^ with later identified homozygous CIB1 deficiency^7^. In contrast to the human phenotype, mice with Cib1 deficiency showed prolonged bleeding, impaired thrombus formation and hemostasis as well as male sterility^48,49^. The reason for the phenotypical variances between human and mice could be related to a different function of the protein in these species and has to be investigated in further studies.

In conclusion, CIB1 deficiency results in few changes in gene expression in the in vitro model developed in the presented study consistent with the clearly defined phenotype in patients^7^. These observations support the hypothesis of human CIB1 as a restriction factor for β-HPV and indicate that, in case of a *CIB1* deficiency, other hypothetical functions of CIB1 can be compensated. The presented model can be used in future studies to investigate CIB1 interaction with β-HPV as well as influence on viral entry or proliferation.

## Supplementary information

Supplementary Information 1.

## Data Availability

Sequence reads of the RNA-seq experiment can be accessed in the European Nucleotide Archive (ENA) under the accession number "PRJEB34355".
