# Supplementary material for: Gene expression is stable in a complete CIB1 knockout keratinocyte model
Source: Sci Rep. 2020 Sep 11;10:14952. doi: 10.1038/s41598-020-71889-9 (PMC7486891; doi:10.1038/s41598-020-71889-9)
Supplement: Supplementary file 1 — Supplementary Information 1. [file 41598_2020_71889_MOESM1_ESM.pdf]

Supplementary material to

Gene expression is stable in a complete *CIB1* knockout keratinocyte model

Elias Imahorn[1], Magomet Aushev[2], Stefan Herms[1,3], Per Hoffmann[1,3,4], Sven Cichon[1,4], Julia Reichelt[5], Peter H. Itin[1,6], Bettina Burger[1,*]

1) Department of Biomedicine, University of Basel and University Hospital Basel, Basel, Switzerland
2) Wellcome Centre for Mitochondrial Research, Institute of Genetic Medicine, Newcastle upon Tyne, UK
3) Institute of Human Genetics, Division of Genomics, Life & Brain Research Centre, University Hospital of Bonn, Bonn, Germany
4) Institute of Neuroscience and Medicine (INM-1), Genomic Imaging, Research Center Juelich, Juelich, Germany
5) Department of Dermatology, Venereology and Allergology, Medical University Innsbruck, Innsbruck, Austria
6) Department of Dermatology, University Hospital Basel, Basel, Switzerland

*) Corresponding author: Bettina Burger, bettina.burger@usb.ch, ORCID: 0000-0002-7686-0176

Supplementary figures

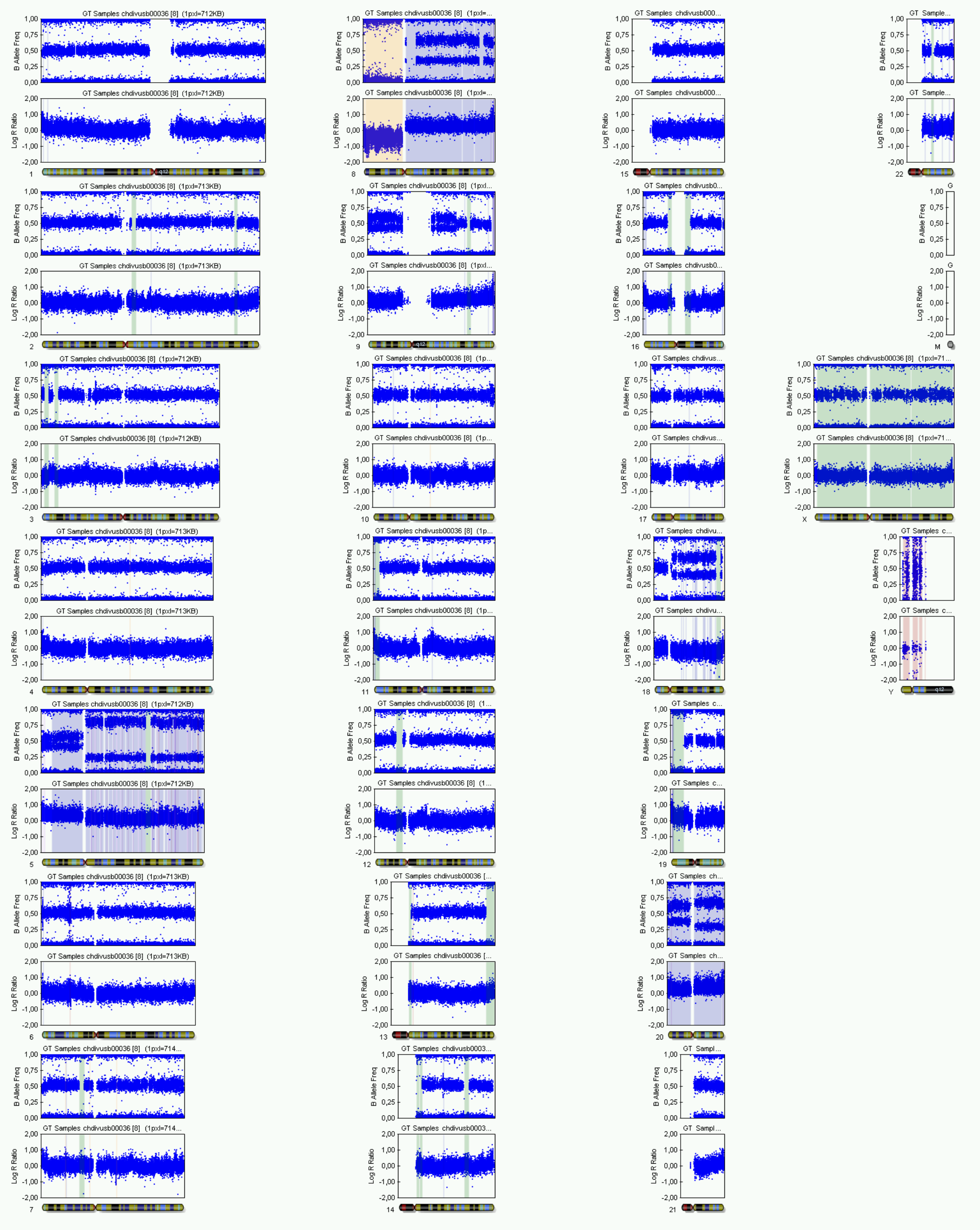

Fig. S1: SNP-array of NKc21 revealed a mostly normal karyotype with a few alterations.

Genomic DNA isolated from NKc21 cells was analyzed by SNP array (Illumina HumanOmniExpress-Exome-8 BeadChip v1.3 SNP array). Data were visualized using GenomeStudio and copy number analysis was performed using CNV-Partition (both Illumina). B allele frequencies and log R ratio of all SNPs on the array were plotted over all human chromosomes for NKc21.

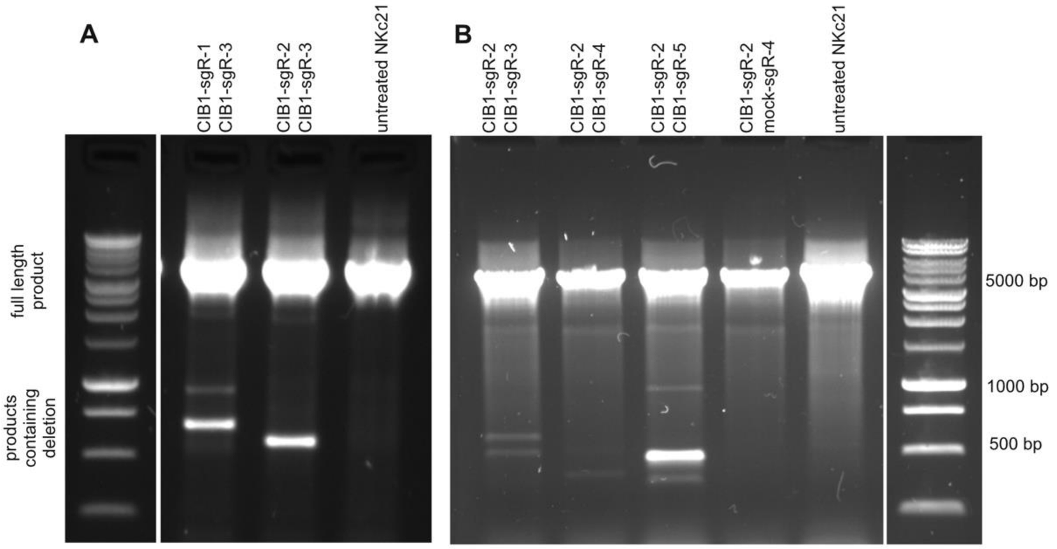

Fig. S2: CRISPR/Cas9 genome editing with pairs of sgRNAs introduces deletions in keratinocyte lines. *CIB1* knockout introduced by two sgRNAs resulted in a shortened amplicon (500–700 bp instead of 4130 bp). For better visibility of the shorter amplicons, these pictures were overexposed resulting in signal saturation of the larger amplicons. A) Combinations CIB1-sgR-1 / CIB1-sgR-3 and CIB1-sgR-2 / CIB1-sgR-3 resulted in the production of shortened amplicons. B) The highest deletion activity was achieved by plasmid combination CIB1-sgR-2 / CIB1-sgR-5. The sgRNA mock-sgR-4 combined with CIB1-sgR-2 has been used as a negative control.

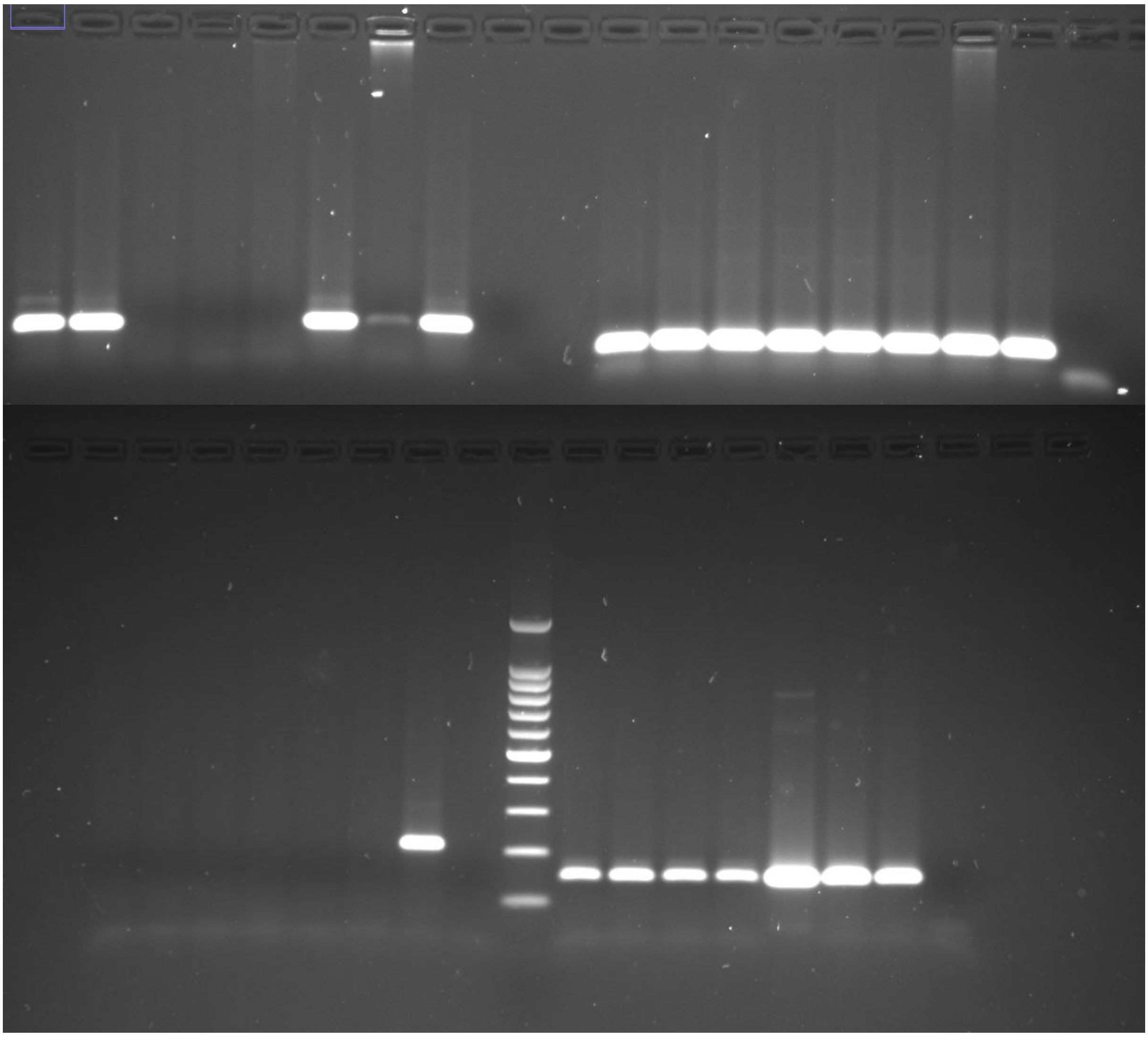

Fig. S3: Expression of *CIB1* mRNA in various clones and untreated NKc21 checked by RT-PCR: Uncropped gel pictures. Uncropped and unlabelled gel pictures that have been used in Figure 1C. Expression of *CIB1* mRNA in various clones and untreated NKc21 was checked by RT-PCR. *LEMD3* was used as a positive control. Nine clones showed no amplification of *CIB1* transcript. Loading scheme upper panel: CIB1 cDNA primer: [clones 2, 6, 26, 34, 35, 42, 44, NKc21 (positive control), H2O (neg ctrl)]; empty lane; LEMD3 cDNA primer: [clones 2, 6, 26, 34, 35, 42, 44, NKc21 (positive control), H2O (neg ctrl)]. Loading scheme lower panel: CIB1 cDNA primer: [clones 50, 53, 63, 65, 66, 72, NKc21 (positive control), H2O (neg ctrl)]; DNA size ladder; LEMD3 cDNA primer: [clones 50, 53, 63, 65, 66, 72, NKc21 (positive control), H2O (neg ctrl)].

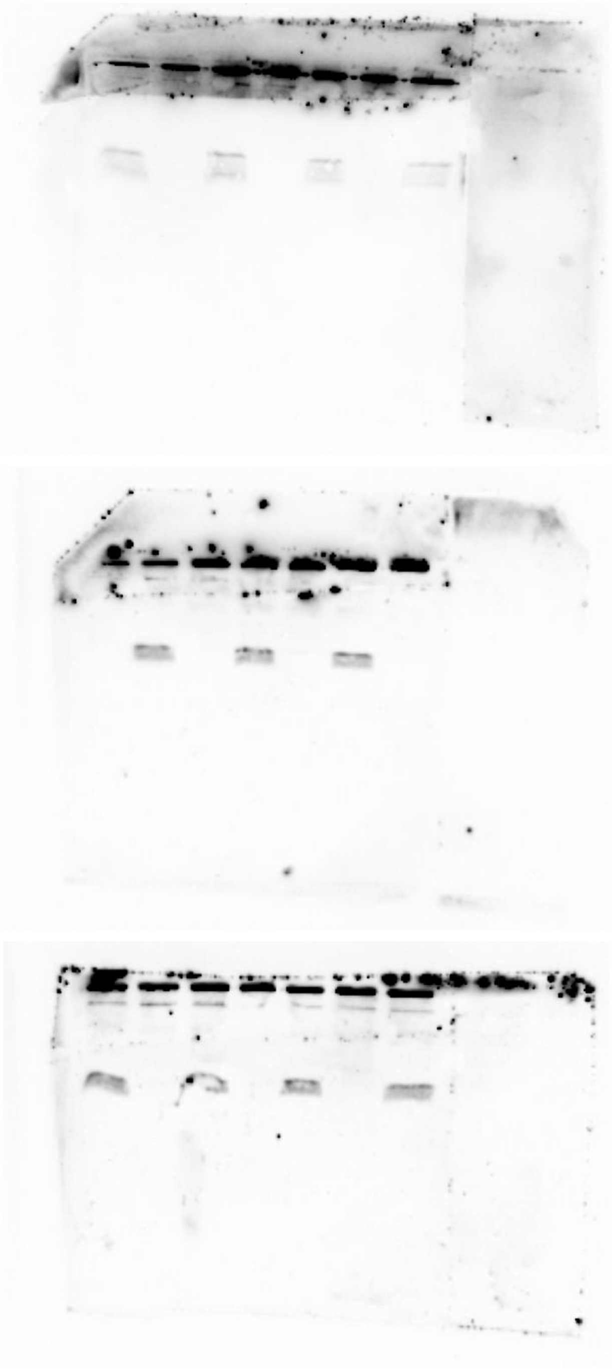

Fig. S4: CIB1 protein expression in various clones and untreated NKc21 checked by Western blot: Uncropped blot pictures.

Uncropped and unlabelled blot pictures that have been used in Figure 1D. Expression of CIB1 protein in various clones and untreated NKc21 was checked by Western blot. Vinculin was used as a positive control. Nine clones showed no CIB1 expression. In all blots, the upper part has been stained with vinculin antibody and the lower part with CIB1 antibody.

Loading scheme blot 1: clones m1, 26, m2, 34, m3, 50, m4; negative controls (no primary antibodies): clones m1, 26

Loading scheme blot 2: clones 35, m5, 53, m6, 62, m7, 65; negative controls (no primary antibodies): clones 35, m5

Loading scheme blot 3: clones m8, 66, m9, 72, m1, 26, NKc21 (pos. control); negative controls (no primary antibodies): clones m8, 66

Table S1: Sequences of oligonucleotides used as PCR or sequencing primers.

Primer name	Sequence
CIB1_1	5'-GATGCACCTCGCGACTTTC-3'
CIB1_2	5'-CCGAAGCTGTCTCTAGAGG-3'
CIB1_3	5'-GGCTCCTTTCCCAACTTCTT-3'
CIB1_4	5'-ATCCCAAAGCTAGTGGCAGA-3'
CIB1_5	5'-TCCTGTGACCTGCTCTCTCA-3'
CIB1_6	5'-GAAGGGGAGAGGTCTTGACA-3'
CIB1_7	5'-CATAGCCCGTTCCAAAGAAG-3'
CIB1_8	5'-CCTCTGCTCGATATGCTGCT-3'
CIB1_9	5'-CCCTTCGAGCAGATTCTCAG-3'
CIB1_10	5' TCAAGGTTCCGTCATCATCA 3'
LEMD3_1	5'-TGCCTATTCCACATGTACGC-3'
LEMD3_2	5'-CCAGAAAATCTGCACCACCT-3'
U6	5'-GAGGGCCTATTTCCCATGATTCC-3'
TMC6_1	5'-TGTACCGGTTCCTGGTGATGGACT-3'
TMC6_2	5'-TTTCCATCAGGTACCGGTGCA-3'
TMC8_1	5'-CTCTTCGGCACAGGAATTCGGTC-3'
TMC8_2	5' GCTGTACACGGAGCTGCTCTC-3'

Table S2: CRISPR/Cas9 target site sequences and predicted double strand break location in CIB1 (NM_006384.3).

sgRNA ID	target sequence without PAM (always NGG)	location of double strand break (NM_006384.3)
**CIB1-sgR-1**	GCGTCACTGCCCGGTCCCCG	c.-91_-90
**CIB1-sgR-2**	GGCGAGCTGCCGGCTCCAAG	c.-190_-189
**CIB1-sgR-3**	GCTGAACAAGAGAAAAGCGG	c.555-17_555-16
**CIB1-sgR-4**	AGGGGCGGCCCTCGTTGTCA	c.*156_*157
**CIB1-sgR-5**	TTGGCCCGCACTGGCAACAC	c.*78_*79
**mock-sgR-1**	GCACTACCAGAGCTAACTCA	no target on the human genome
**mock-sgR-2**	TCTCGACAATCTATCGAGCG	no target on the human genome
**mock-sgR-3**	AGAGTGGGCGCGAATAACGC	no target on the human genome
**mock-sgR-4**	TAAGCAGCATATCGAAGCAG	no target on the human genome
